# Supplementary material for: Maternal age-dependent APC/C-mediated decrease in securin causes premature sister chromatid separation in meiosis II
Source: Nat Commun. 2017 May 18;8:15346. doi: 10.1038/ncomms15346 (PMC5454377; doi:10.1038/ncomms15346)
Supplement: Supplementary Information — Supplementary Figures [file ncomms15346-s1.pdf]

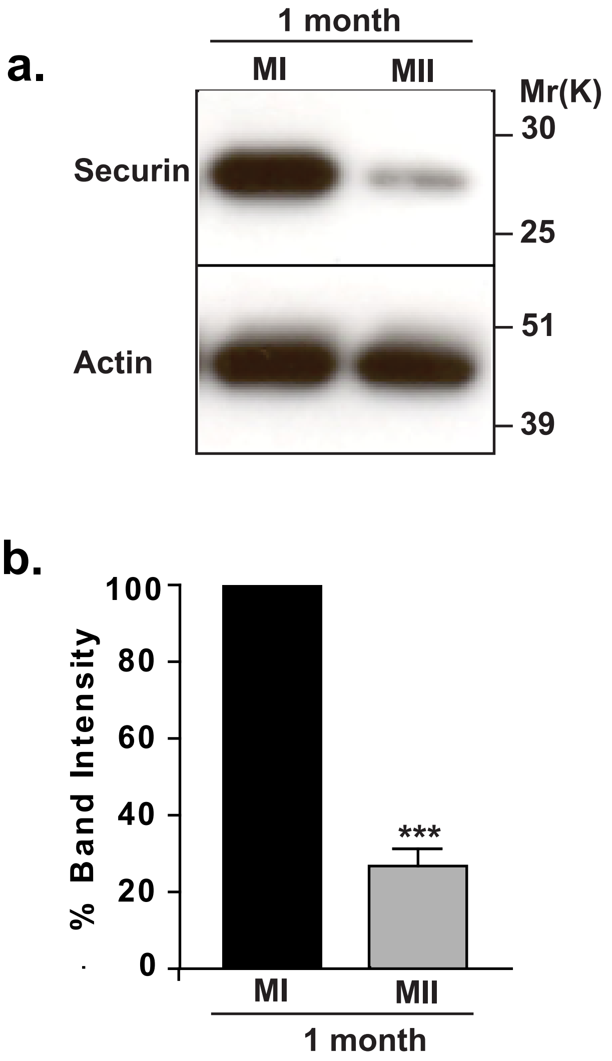

Supplementary Figure 1: Failure of endogenous securin to re-accumulate following MI exit in oocytes from young mice. **(a)** Western blot and **(b)** densitometric analysis of oocytes (30 oocytes per lane) from 1 month-old mice for securin during MI (8 h post release from GV arrest) and MII arrest (14 h post release). Actin was used as a loading control. Results are mean + SEMs. ***P<0.001. P value was calculated with one-sided Student's *t*-test. Results are representative of three independent experiments involving three mice per experimental group .

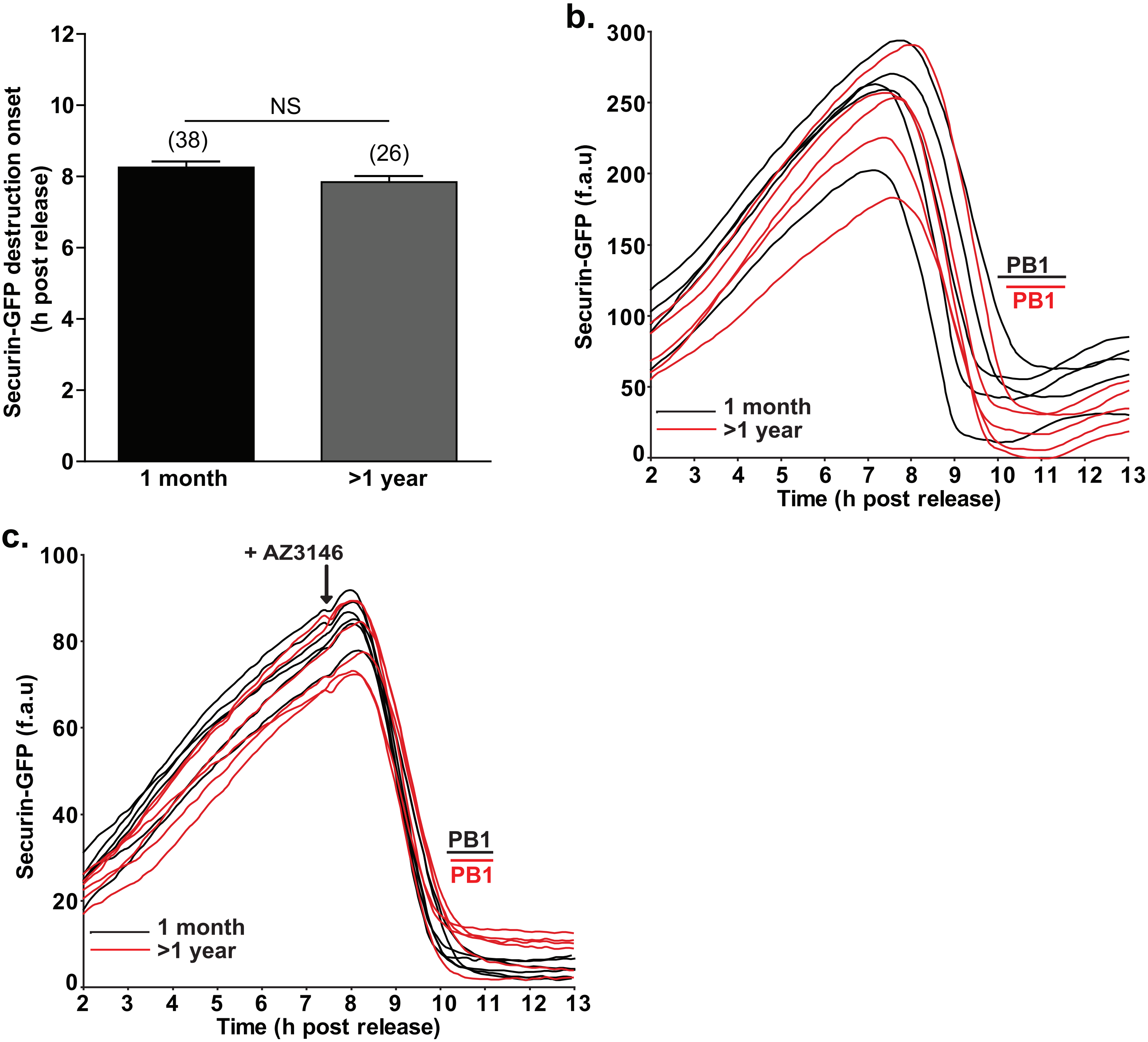

Supplementary Figure 2: Securin-GFP kinetics in oocytes from young and aged mice. (a) Meantime of securin-GFP destruction onset in oocytes from 1 month- and >1 year-old mice. The number of oocytes used is shown in parentheses. Results are mean + SEMs. $^{NS}P>0.05$. P value was calculated with one-sided Student's *t*-test. (b, c) Representative fluorescence traces of securin-GFP and period over which PB1 extrusion occurs in oocytes from 1 month- and >1 year-old mice, in absence (b) or presence (c) of AZ3146. In c, the inhibitor was added at 7.5 h post release from GV arrest (arrow). Results are from two to three independent experiments involving two to five mice per experimental group.

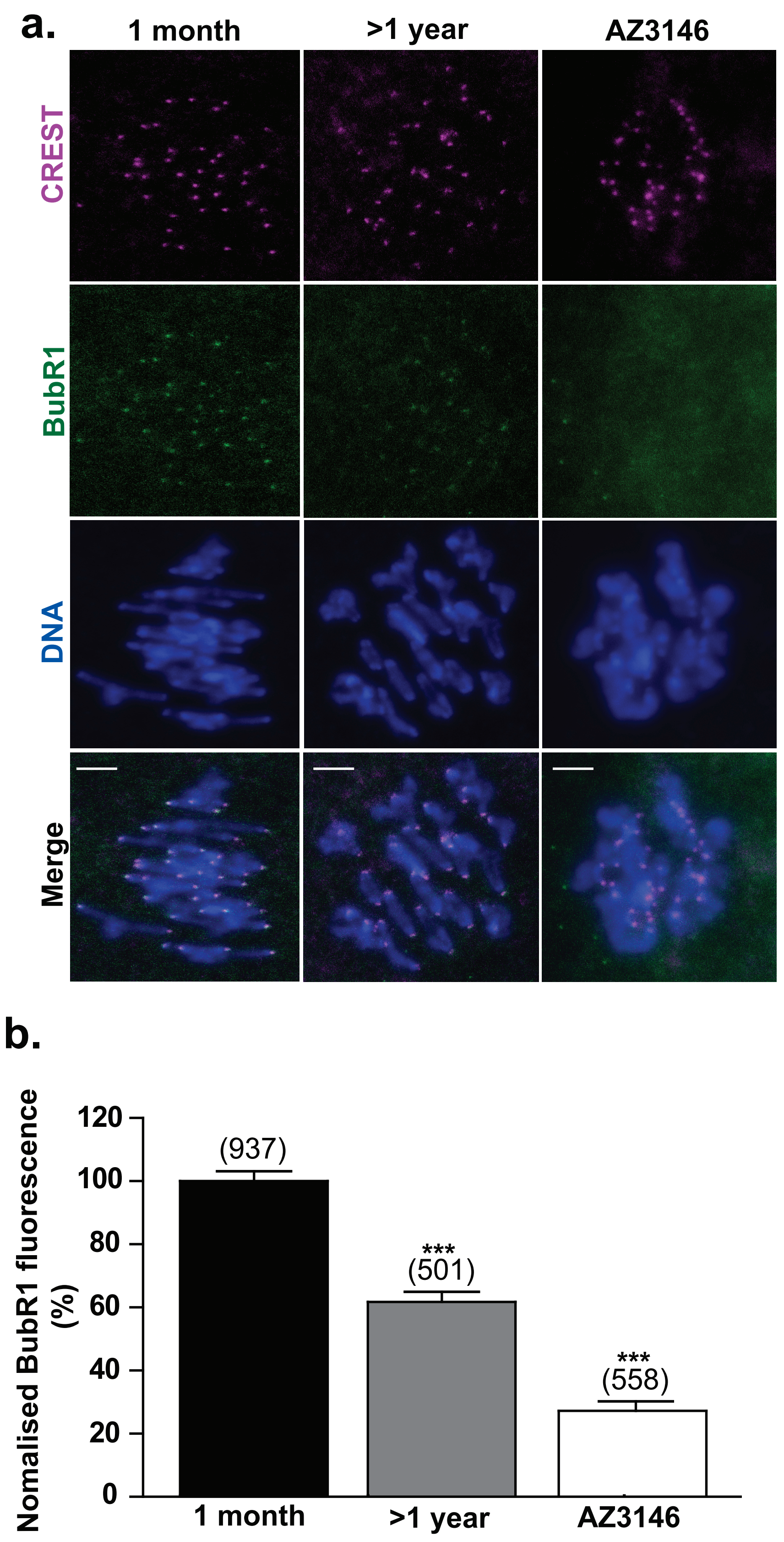

Supplementary Figure 3: Compromised SAC in oocytes from aged mice at the MI-to-MII transition. (a) Representative images of immunostaining and **(b)** quantification of BubR1-kinetochore fluorescence in oocytes from 1 month (n=25 oocytes) and >1 year (n=15 oocytes) old mice, as well as oocytes (n=18 oocytes) from 1 month-old mice that were treated with AZ3146 from 7.5 h post release from GV. All oocytes were fixed at 11 h post release, just prior to PB1. DNA is shown in blue, BubR1 in green and CREST in magenta. The number of sister-kinetochores measured is shown in parentheses. Bars, 10 μm. Results are mean + SEMs. ***P<0.001. P values were calculated with one-sided Student's *t*-test. Results are representative of three independent experiments involving two to five mice per experimental group.

Securin-GFP

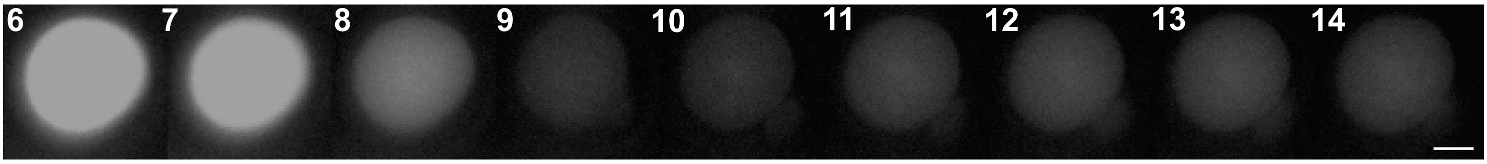

Supplementary Figure 4: Injection of securin-GFP cRNA into GV-stage oocytes leads to detectable securin-GFP fluorescence in MII-stage oocytes. Representative securin-GFP images of an oocyte from >1year-old mouse injected with securin-GFP cRNA at the GV stage. Times indicated are in hours after GV release. Bar, 20 μm.

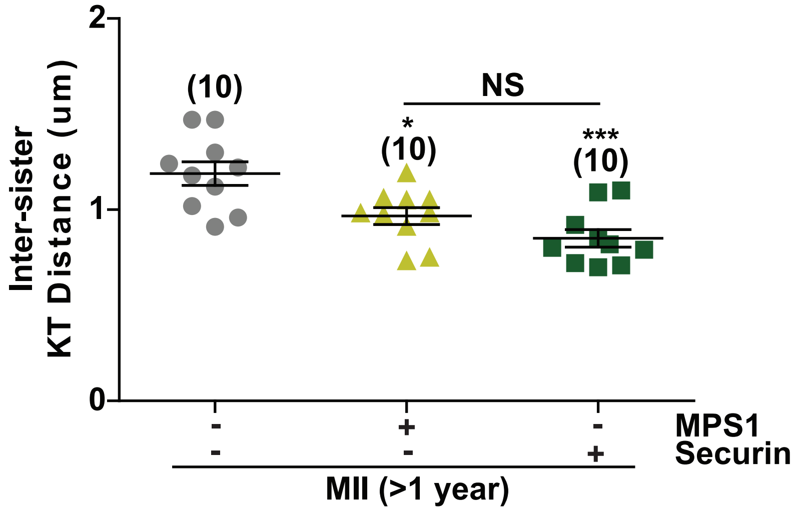

Supplementary Figure 5: Mean inter-sister kinetochore distances within individual MII eggs. All MII oocytes are from >1year old mice. The oocytes were microinjected with either Mps1-GFP or securin-GFP cRNA at the GV stage and fixed at 14 h post release from GV arrest. The number of oocytes used is shown in parentheses. Results are mean ± SEMs. ^{NS}P>0.05, *P<0.05 and ***P<0.001. P values were calculated with one-sided Student's *t*-test. Results are from two to four independent experiments involving two to five mice per experimental group.

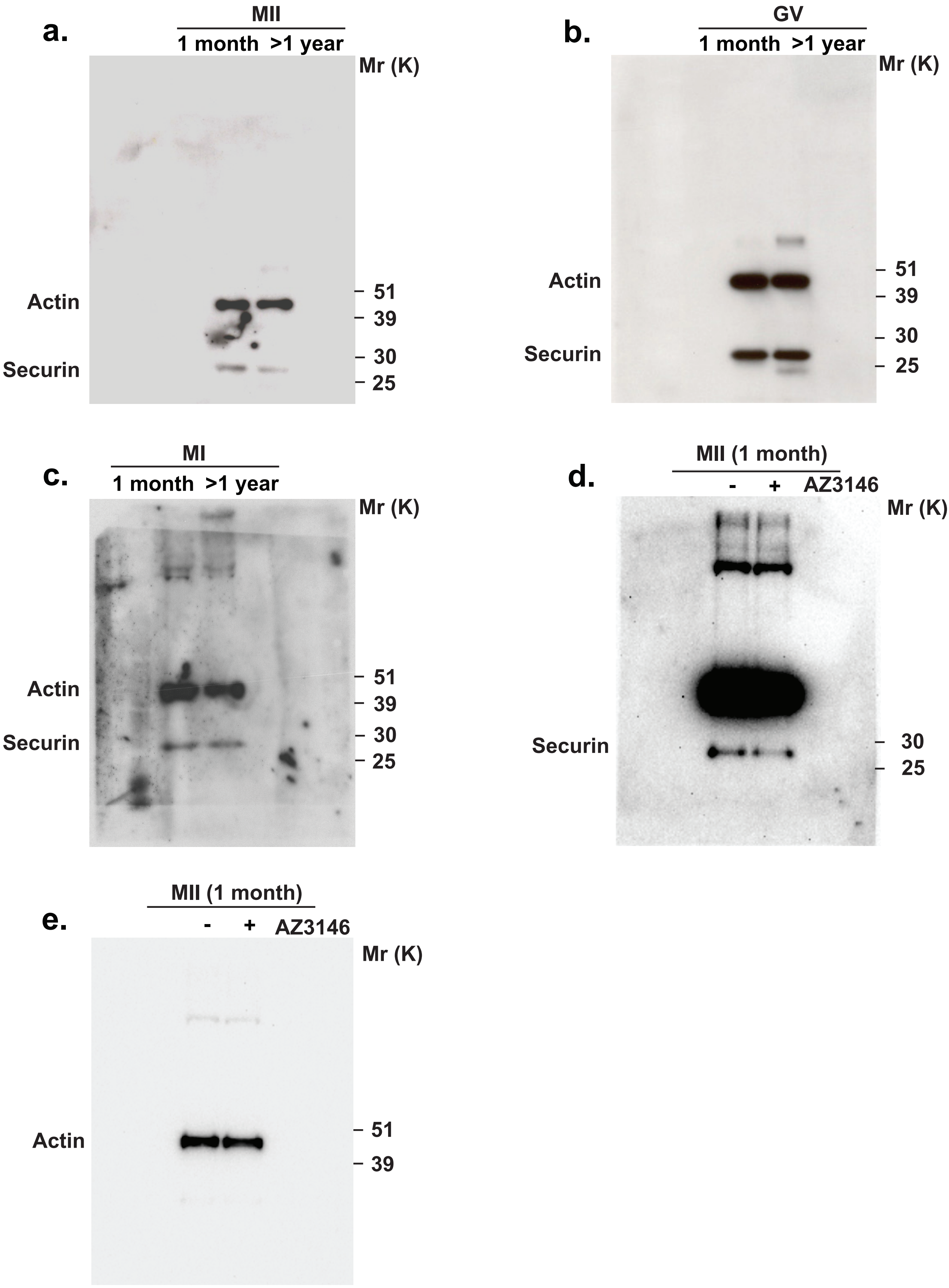

Supplementary Figure 6: Whole gel images of Western blots from figures 2 and 3. **(a)** Figure 2a. **(b)** Figure 2c. **(c)** Figure 2e. **(d, e)** Figure 3h.
